# Supplementary material for: Causal interplay between lactose intolerance and gut microbiota: a combined bidirectional Mendelian randomization and in vivo validation study
Source: Front Nutr. 2026 Jun 1;13:1803337. doi: 10.3389/fnut.2026.1803337 (PMC13265576; doi:10.3389/fnut.2026.1803337)
Supplement: Supplementary file 1 [file Data_Sheet_1.zip › supplementary materials/Forward/forest plot/ebi-a-GCST90027769.finngen_R12_E4_LACTONAS.pdf]

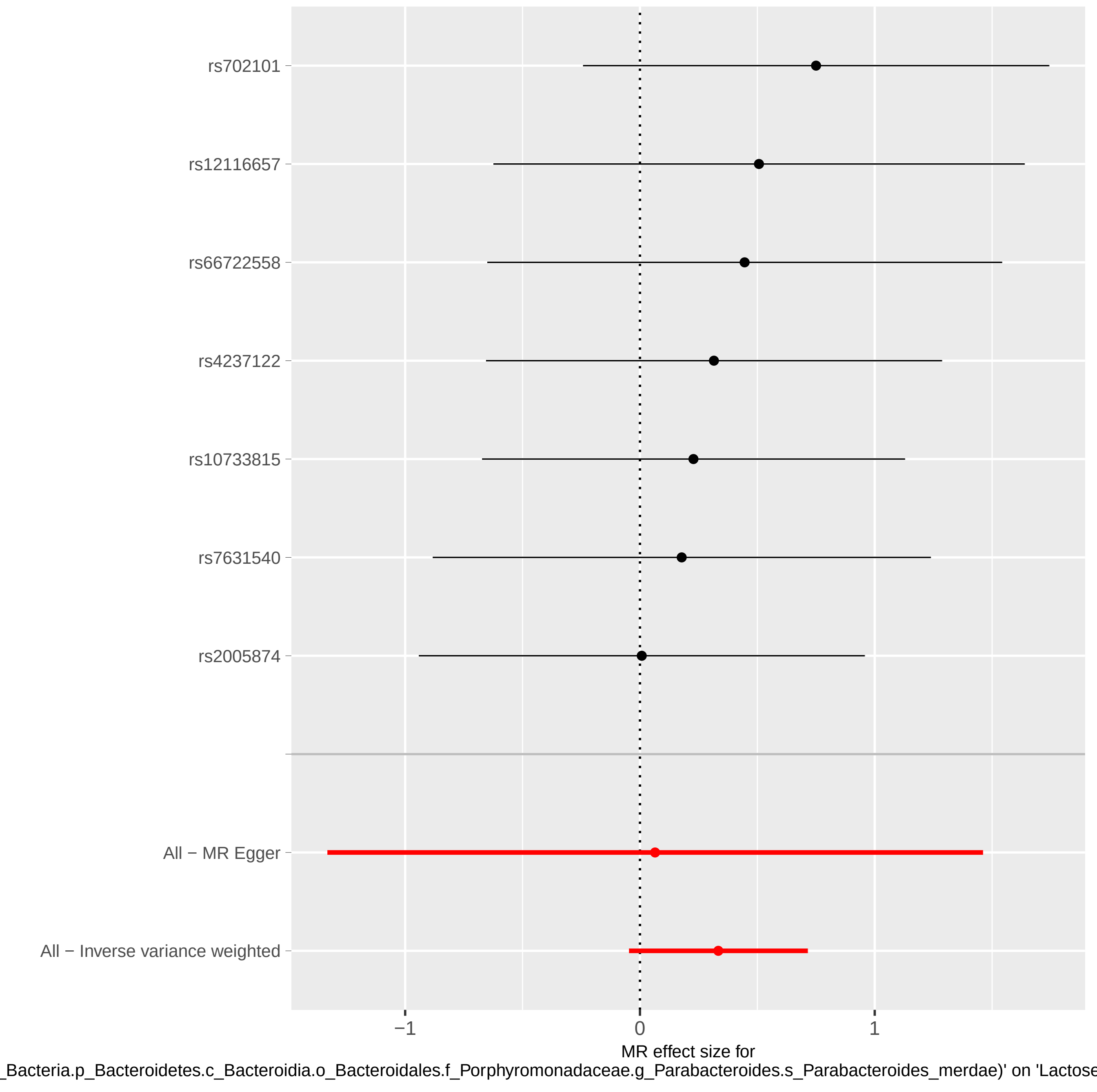
rs702101
rs12116657
rs66722558
rs4237122
rs10733815
rs7631540
rs2005874
All − MR Egger
All − Inverse variance weighted
−1
0
1
MR effect size for
_Bacteria.p_Bacteroidetes.c_Bacteroidia.o_Bacteroidales.f_Porphyromonadaceae.g_Parabacteroides.s_Parabacteroides_merdae)' on 'Lactose
